# Machine learning-based survival prediction nomogram for postoperative parotid mucoepidermoid carcinoma

**DOI:** 10.1038/s41598-024-58329-8

**Published:** 2024-04-01

**Authors:** Zongwei Huang, Zihan Chen, Ying Li, Ting Lin, Sunqin Cai, Wenxi Wu, Lishui Wu, Siqi Xu, Jun Lu, Sufang Qiu

**Affiliations:** 1 Fujian Medical University Cancer Hospital, Fujian Cancer Hospital, Fuzhou, China; 2 Radiation Oncology Department, Clinical Oncology School, Fujian Cancer Hospital, Fujian Medical University, Fuzhou, Fujian China

**Keywords:** Machine learning, Parotid mucoepidermoid carcinoma, Nomogram, Postoperative radiotherapy, SEER, Head and neck cancer, Risk factors

## Abstract

Parotid mucoepidermoid carcinoma (P-MEC) is a significant histopathological subtype of salivary gland cancer with inherent heterogeneity and complexity. Existing clinical models inadequately offer personalized treatment options for patients. In response, we assessed the efficacy of four machine learning algorithms vis-à-vis traditional analysis in forecasting the overall survival (OS) of P-MEC patients. Using the SEER database, we analyzed data from 882 postoperative P-MEC patients (stages I–IVA). Single-factor Cox regression and four machine learning techniques (random forest, LASSO, XGBoost, best subset regression) were employed for variable selection. The optimal model was derived via stepwise backward regression, Akaike Information Criterion (AIC), and Area Under the Curve (AUC). Bootstrap resampling facilitated internal validation, while prediction accuracy was gauged through C-index, time-dependent ROC curve, and calibration curve. The model’s clinical relevance was ascertained using decision curve analysis (DCA). The study found 3-, 5-, and 10-year OS rates of 0.887, 0.841, and 0.753, respectively. XGBoost, BSR, and LASSO stood out in predictive efficacy, identifying seven key prognostic factors including age, pathological grade, T stage, N stage, radiation therapy, chemotherapy, and marital status. A subsequent nomogram revealed a C-index of 0.8499 (3-year), 0.8557 (5-year), and 0.8375 (10-year) and AUC values of 0.8670, 0.8879, and 0.8767, respectively. The model also highlighted the clinical significance of postoperative radiotherapy across varying risk levels. Our prognostic model, grounded in machine learning, surpasses traditional models in prediction and offer superior visualization of variable importance.

## Introduction

Parotid gland carcinoma represents an uncommon, markedly heterogeneous malignancy of the head and neck region^1^. As per the World Health Organization (WHO) classification, over 20 histological subtypes exist, encompassing adenoid cystic carcinoma, mucoepidermoid carcinoma (MEC), secretory carcinoma, among others, with MEC being the predominant tissue type^2^. MEC is characterized as a malignant glandular epithelial neoplasm, characterized by the presence of mucinous, intermediate, and epidermoid cells exhibiting columnar, clear cell, and cancer-like features. Based on invasiveness and differentiation, MEC is stratified into low, intermediate, and high-grade malignant neoplasms^3–5^.

The tumor’s pathological grade is an important factor in determining the prognosis for MEC patients, and it often guides treatment approaches^6–9^. However, the TNM system and pathological grade fall short in estimating survival or informing adjuvant therapy-related decisions^10^. Previous research has shown that high-grade tumors and those with positive margins are at a higher risk of recurrence and spread. Post-operative radiation therapy can help to prevent the recurrence of the tumor and improve overall outcomes for these patients^11–13^. Therefore, postoperative management is crucial for the long-term prognosis of parotid mucoepidermoid carcinoma (P-MEC) patients. Additionally, the postoperative stage is also a critical period for disease monitoring and making clinical decisions, such as the development of postoperative radiation therapy plans. Hence, a comprehensive and accurate postoperative survival prediction model is needed to integrate multiple clinical pathological features to guide treatment decision-making and disease monitoring.

Although the preoperative phase and initial treatment strategies are undoubtedly important in the management of P-MEC, the majority of patients undergo surgery as the primary treatment modality. Therefore, the objective of this study is to establish a personalized, clinically valuable, and effective predictive model specifically for postoperative P-MEC. Prior research has been limited by small sample sizes, brief study durations, and single-center designs. In contrast, the Surveillance, Epidemiology, and End Results (SEER) database serves as a comprehensive trove of clinical data collected from a diverse array of cancer patients across multiple registries spanning the vast expanse of the United States. The database’s wide-reaching coverage imbues it with the capacity to offer a representative sample of cancer incidence and survival rates across the entire US populace, thereby enhancing the credibility and robustness of research studies^10^. Machine learning methods have been extensively employed in other tumor prediction models, with their analytical and visual capabilities serving as potent tools for clinical forecasting. Machine learning approaches consider a broader range of variable relationships compared to conventional statistical methodologies. To date, no studies have explored machine learning-based predictive models for postoperative P-MEC^14,15^.

A nomogram integrating visual representations with hazard probability scores for each prognostic factor to construct a proportional hazards regression model was developed and validated using a large SEER population database and screening suitable variables via traditional and machine learning methods. Moreover, risk stratification based on the nomogram scores substantially enhances clinical applicability, offering clinicians tailored treatment strategies for patients with postoperative P-MEC.

## Method

### Study population: SEER data

**Figure 1:**
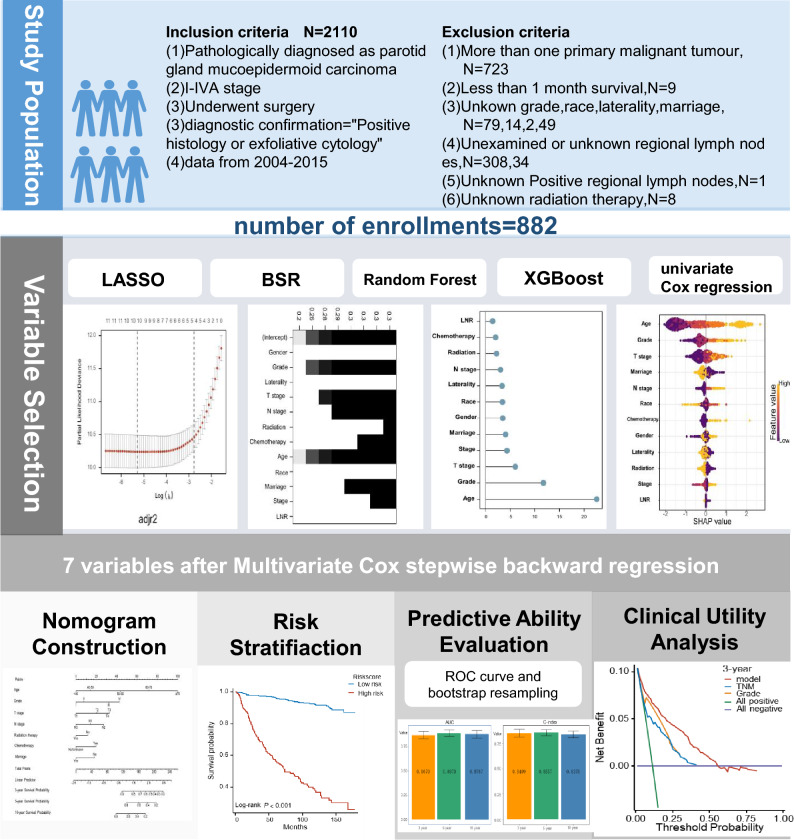
Analysis flow for the development and evaluation of models for parotid mucoepidermoid carcinoma(P-MEC).

For this study, SEER^*^STAT software (version 8.4.0) was employed to extract clinical data of patients with P-MEC between 2004 and 2015.The criterias for the inclusion of P-MEC patients were as follows: (1) patients with a primary site of “parotid gland: 07.9”; (2) patients with pathologically confirmed stage I-IVA P-MEC; (3) post-surgical patients; (4) patients with diagnostic confirmation of “Positive histology or exfoliative cytology”. Additionally, patients who were not primary cases, lived less than 1 month, had unclear basic information, were not examined for regional lymph nodes, or had inaccurate positive lymph node results were excluded. The screening process is illustrated in Fig. 1.

### Variable selection

To identify the clinical variables that significantly impact the overall survival (OS) of patients, various statistical methods were employed, including extreme gradient boosting (XGBoost), least absolute shrinkage and selection operator (LASSO), best subsets regression (BSR), random forest (RF), and Cox proportional hazard regression. The LNR, representing the ratio of regional nodes testing positive among those examined, was calculated, and its cut-off point was determined using X-tile software. Subsequently, the clinical variables were screened using stepwise backward regression with the “MASS” package. Model fitting was evaluated by the Akaike Information Criterion (AIC), and the area under the receiver operating characteristic curve (AUC) was used to evaluate the model’s accuracy. The ‘survival’ package was used for proportional hazards hypothesis testing and Cox regression analysis, the ‘caret’ package for RF analysis with default settings and 20-fold cross-validation, the ‘shapviz’ and ‘xgboost’ packages for XGBoost analysis, the ‘glmnet’ package for LASSO analysis, and the ‘leaps’ package for BSR analysis.

### Nomogram construction

The nomogram, an effective visual tool for predicting survival outcomes, was constructed using the “rms,” “timeROC,” and “nomogramFormula” packages, based on the variables included in the Cox proportional hazards regression model. The nomogram values were assigned to each patient, and the ROC curve analysis was utilized to determine the optimal cutoff value for risk stratification.

### Model validation

The calibration curves were used to assess the agreement between the predicted probabilities and observed outcomes, and the ROC curve and AUC were utilized to evaluate the model’s discrimination ability. The internal validation was performed using the bootstrapping method, where 1000 resampling was performed to validate the model, using the “riskRegression” and “pec” packages^16^. Moreover, to identify the clinical utility of the model, the decision curve analysis (DCA) through the “stdca.R” package was employed^17^. Additionally, the Kaplan–Meier (K–M) method was used to compare the OS of various groups, and the log-rank test was used to evaluate its significance with a P-value threshold of < 0.05. The statistical analyses were performed using R software (R version 4.2.1), and the seed was set to 1234 for all the tests.

## Results

### Screening and characteristics of the patients

This study examined 882 patients with stage I–IVA P-MEC, who met the inclusion–exclusion criteria, from the SEER database between 2004 and 2015. Figure 1 illustrates the patient selection process, while Table 1 summarizes patients’ demographic and clinicopathological characteristics. The lymph node ratio (LNR) cut-off was determined using X-tile analysis, with a resultant cut-off of 1.15%. The median (95% CI) follow-up time was 99 (92–105) months, and the median (IQR) age at diagnosis was 52 (37–66) years. A majority of the patients were white (661, 74.9%), with most tumors being grade II (396, 44.9%), stage I (353, 40%), T1-stage (381, 43.2%), N0-stage (685, 77.7%), and LNR0 (686, 77.8%) according to the AJCC 6th stage. All variables, except for chemotherapy (94.2% vs 5.8%), had proportions exceeding 10%. The study encompassed 12 variables, including age, gender, grade, stage, tumor (T) stage, node (N) stage, radiation, chemotherapy, laterality, marriage, and LNR. Nine factors—age, gender, grade, stage, T stage, N stage, radiation, chemotherapy, and LNR—were selected based on univariate Cox regression. Multivariate Cox regression revealed that four factors (age, grade, T stage, and chemotherapy) were independent risk factors, each with P-values less than 0.05. In the multivariate analysis, individuals aged 60–70 years (HR = 5.936, 95% CI = 3.016–11.681, P < 0.001), those over 70 years old (HR = 11.962, 95% CI = 6.303–22.703, P < 0.001), Grade III (HR = 2.324, 95% CI = 1.235–4.375, P = 0.009), Grade IV (HR = 3.148, 95% CI = 1.710–5.795, P < 0.001), T2 (HR = 3.162, 95% CI = 1.059–9.440, P = 0.039), T3 (HR = 4.300, 95% CI = 1.501–12.316, P = 0.007), T4 (HR = 4.414, 95% CI = 1.439–13.535, P = 0.009), and chemotherapy (HR = 1.721, 95% CI = 1.096–2.703, P = 0.018) emerged as independent risk factors for overall survival (OS). Nevertheless, radiation(HR = 0.750, 95% CI = 0.525–1.072, P = 0.114), LNR (HR = 0.868, 95% CI = 0.114–6.602, P = 0.891), and other variables demonstrated no prognostic value (Table 2).

**Table 1:** Demographic and clinical characteristics of patients with P-MEC.

Characteristics	n (%)
Age	
Median (IQR)	52.00 [37.00, 66.00]
< 40	254 (28.8)
40–50	146 (16.6)
50–60	173 (19.6)
60–70	138 (15.6)
≥ 70	171 (19.4)
Gender	
Female	445 (50.5)
Male	437 (49.5)
Race	
White	669 (74.9)
Black	120 (13.6)
Other	101 (11.5)
Grade	
I	234 (26.5)
II	396 (44.9)
III	120 (13.6)
IV	132 (15)
Stage	
I	353 (40)
II	202 (22.8)
III	170 (18.9)
IVA	161 (18.3)
Tumor (T) stage	
T1	381 (43.2)
T2	257 (29.1)
T3	145 (16.4)
T4	99 (11.2)
Node (N) stage	
N0	685 (77.7)
N1	110 (12.5)
N2	87 (9.9)
Radiation	
No	450 (51)
Yes	432 (49)
Chemotherapy	
No/unknown	831 (94.2)
Yes	51 ( 5.8)
Laterality	
Left	413 (46.8)
Right	469 (53.2)
Marriage	
No	390 (44.2)
Yes	492 (55.8)
Lymph node ratio (%)	
Median (IQR)	0.00 [0.00, 0.00]
LNR0	686 (77.8)
LNR1	196 (22.2)

Grade is codefined as follows: Grade I, well differentiated, Grade II, moderately differentiated; Grade III, poorly differentiated, Grade IV, undifferentiated; anaplastic.

Lymph node ratio, the number of positive lymph nodes divided by the number of neck lym-ph nodes dissected. LNR0, LNR(%) < 1.15. LNR1, LNR(%) ≥ 1.15.

*IQR,* inter quartile range.

**Table 2:** Univariate and multivariate analyses for OS in patients with P-MEC.

Characteristics	Univariable analysis		Multivariable analysis	
	HR (95% CI)	P-value	HR (95% CI)	P-value
Age		** < 0.001**		
< 40	Reference		Reference	
40–50	1.453 (0.628–3.363)	0.383	1.305 (0.561–3.036)	0.536
50–60	3.282 (1.656–6.505)	< 0.001	2.594 (1.286–5.234)	0.008
60–70	7.195 (3.766–13.746)	< 0.001	5.936 (3.016—11.681)	** < 0.001**
≥ 70	18.925 (10.379—34.508)	< 0.001	11.962 (6.303—22.703)	** < 0.001**
Gender		** < 0.001**		
Female	Reference		Reference	
Male	2.112 (1.563–2.855)	< 0.001	1.101 (0.797–1.522)	0.560
Race		** < 0.001**		
White	Reference		Reference	
Black	0.533 (0.319–0.891)	0.016	1.444 (0.840–2.483)	0.184
Other	0.437 (0.237–0.806)	0.008	0.839 (0.449–1.570)	0.584
Grade		** < 0.001**		
I	Reference		Reference	
II	1.668 (0.975–2.856)	0.062	1.346 (0.773–2.343)	0.294
III	6.837 (3.981–11.741)	< 0.001	2.324 (1.235–4.375)	**0.009**
IV	9.505 (5.654–15.978)	< 0.001	3.148 (1.710–5.795)	** < 0.001**
Stage		** < 0.001**		
I	Reference		Reference	
II	1.668 (1.029–2.706)	0.038	0.481 (0.145–1.596)	0.232
III	3.353 (2.173–5.175)	< 0.001	0.480 (0.149–1.544)	0.218
IVA	5.950 (3.970–8.917)	< 0.001	0.462 (0.130–1.638)	0.232
Tumor (T) stage		** < 0.001**		
T1	Reference		Reference	
T2	2.199 (1.454–3.325)	< 0.001	3.162 (1.059–9.440)	**0.039**
T3	4.210 (2.773–6.390)	< 0.001	4.300 (1.501–12.316)	**0.007**
T4	5.440 (3.534–8.376)	< 0.001	4.414 (1.439- 13.535)	**0.009**
Node (N) stage		** < 0.001**		
N0	Reference		Reference	
N1	2.972 (2.067–4.274)	< 0.001	1.807 (0.226–14.438)	0.577
N2	4.849 (3.400–6.916)	< 0.001	2.517 (0.300–21.156)	0.395
Radiation		** < 0.001**		
No	Reference		Reference	
Yes	2.074 (1.531–2.810)	< 0.001	0.750 (0.525–1.072)	0.114
Chemotherapy		** < 0.001**		
No/unknown	Reference		Reference	
Yes	3.871 (2.569–5.832)	< 0.001	1.721 (1.096–2.703)	**0.018**
Laterality		0.648		
Left	Reference			
Right	0.935 (0.702–1.246)	0.648		
Marriage		0.327		
No	Reference			
Yes	0.866 (0.649–1.154)	0.326		
Lymph node ratio 9%)		** < 0.001**		
LNR0	Reference		Reference	
LNR1	3.647 (2.731–4.869)	< 0.001	0.868 (0.114–6.602)	0.891

Statistical significance indicated by P < 0.05 has been denoted in bold.

### Variable selection

**Figure 2:**
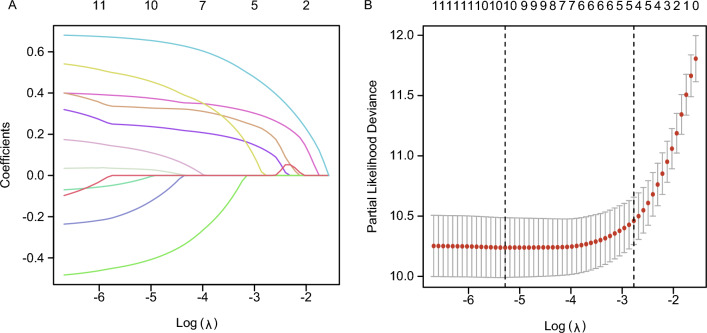
Predictor Screening: the least absolute shrinkage and selection operator (LASSO) regression and fivefold cross-validation.

**Figure 3:**
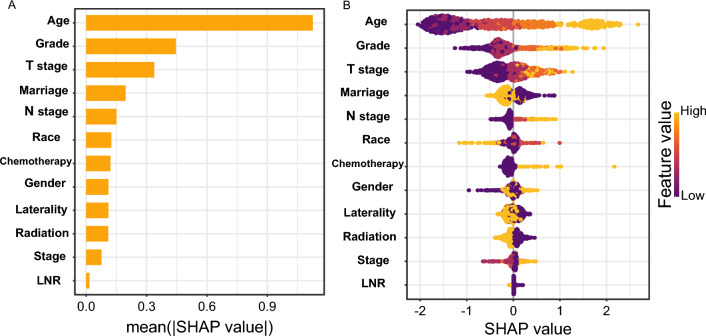
Predictor Screening: A SHAP plot and a feature importance plot are visualizations used to interpret XGBoost model results.

**Figure 4:**
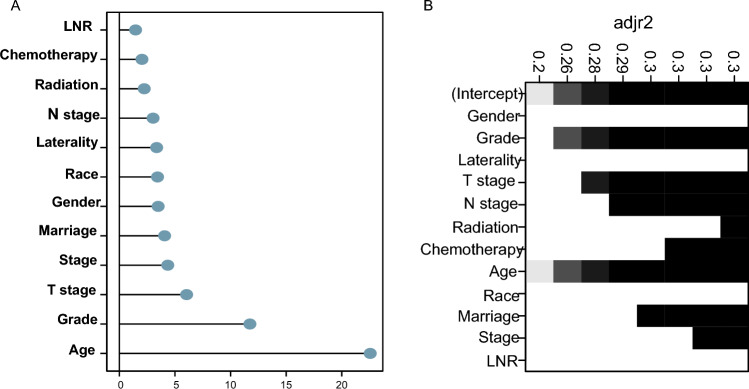
Predictor Screening: (**A**) Random Forest importance plot; (**B**) Best Subset Regression (BSR), it selected the best subset of predictor variables to accurately model a response variable.

Figure 2A displays the relationship between the LASSO coefficients and the regularization parameter, lambda (λ), and demonstrates the variable selection process and the effect of λ on the coefficients. The “lambda.min” value, which represents the lambda value corresponding to the minimum likelihood deviation or the highest C-index, was utilized for selecting tuning parameters in LASSO regression. Another vertical line was “lambda.1se,” which corresponds to the most regularized model within one standard error of the minimum (Fig. 2B). The “λ.min” (λ = 0.0050724) was chosen for the best predictive performance. A ten-fold cross-validation was employed. Ten variables were chosen through the LASSO regression algorithm, including age, gender, grade, T stage, N stage, radiation, chemotherapy, laterality, marriage, and LNR. Employing the adjusted R-squared maximum of the BSR, we selected eight variables: age, grade, stage, T stage, N stage, radiation, chemotherapy, and marriage(Fig. 3). In the RF model and XGBoost, we independently extracted the top 10 variables, excluding laterality, radiation (RF), and LNR (XGBoost) (Fig. 4). We assessed the key performance of machine learning and traditional statistics using AUC and AIC. Multivariate Cox stepwise backward regression reconfirmation identified LASSO, BSR, and XGBoost as the best of the five screening methods based on both AUC (AUC = 88.4) and AIC (AIC = 2118.9) criteria (Table 3).

**Table 3:** Screening results of five methods for identifying predictors.

Characteristics	Univariate Cox regression		LASSO regression		Besubset regression		Random forest		XGboost	
	Preliminary	Final	Preliminary	Final	Preliminary	Final	Preliminary	Final	Preliminary	Final
Age	+	+	+	+	+	+	+	+	+	+
Gender	+		+				+		+	
Race	+						+		+	
Grade	+	+	+	+	+	+	+	+	+	+
Stage	+				+		+	+		
T stage	+	+	+	+	+	+	+	+	+	+
N stage	+	+	+	+	+	+	+		+	+
Radiation	+	+	+	+	+	+			+	+
Chemotherapy	+	+	+	+	+	+	+	+	+	+
Laterality			+						+	
Marriage			+	+	+	+	+	+	+	+
Lymph node ratio(%)	+		+				+			
Total number	10	6	10	**7**	8	**7**	10	6	10	**7**
AUC(5 years)		87.7		**88.4**		**88.4**		88.0		**88.4**
AIC	2131.3	2122.3	2123.9	**2118.9**	2122.6	**2118.9**	2129.0	2120.0	2124.2	**2118.9**

Final, the results obtained after the model undergone Multivariate Cox stepwise backward regression.

Statistical significance indicated by P < 0.05 has been denoted in bold.

### Nomogram construction

**Figure 5:**
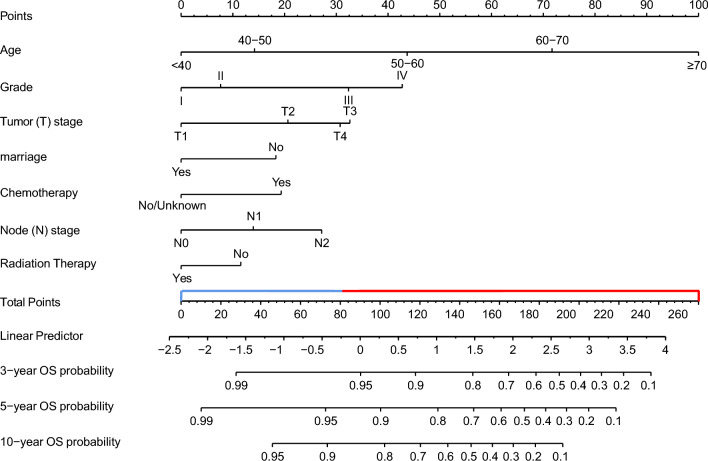
A survival nomogram for predicting overall survival (OS) for patients with P-MEC. (1) When using the nomogram, seven predictors were quantified as “point” based on patient-specific factors and then the sum of the “point” corresponded to the “total point” below, which corresponded to the 3, 5, 10 year OS ; (2) The optimal cut-off total point was 80.29 (the median of patients’ point), which divided the patients into high-risk group and low-risk group.

Consequently, we constructed a nomogram with seven variables from the three algorithms (LASSO, BSR, and XGBoost), including age, grade, tumor stage, node stage, chemotherapy, radiation, and marriage. We developed an OS-nomogram capable of predicting a patient’s 3-, 5-, and 10-year OS rates using these variables (Fig. 5). By converting clinical, pathological, and therapeutic factors into points, the nomogram accurately predicted OS. The total risk point score, calculated by summing all points, significantly correlated with 3-, 5-, and 10-year OS. We utilized a 5-year ROC curve to determine the optimum risk score cut-off point. Kaplan–Meier curves revealed that low-risk group patients (risk score < 80.29) had better survival prognosis compared to high-risk group patients (risk score ≥ 80.29, log-rank test, P < 0.001) (Fig. S1).

### Predictive ability evaluation

**Figure 6:**
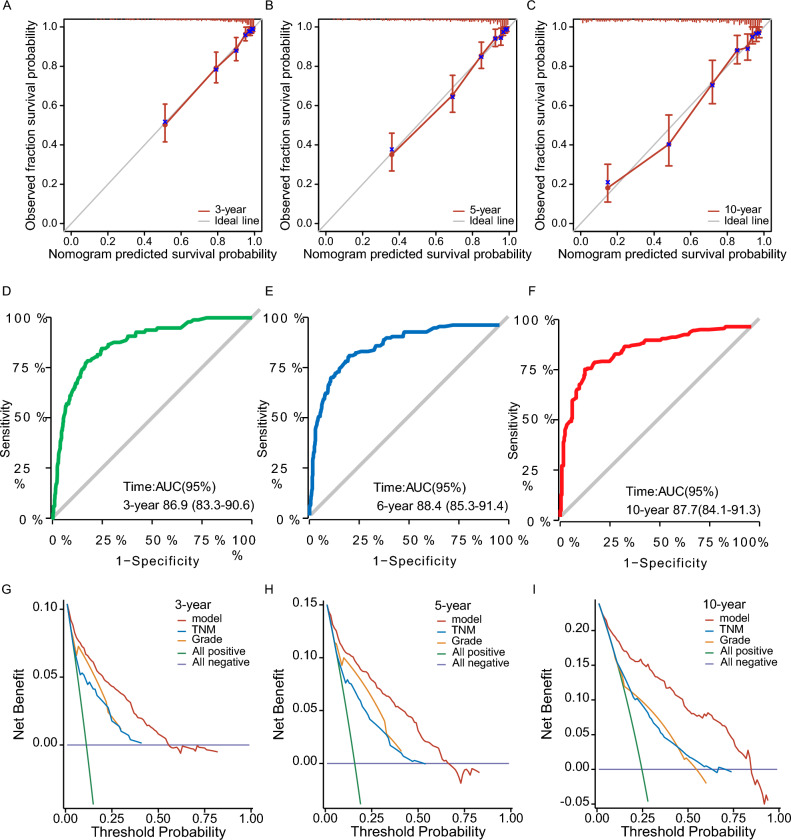
(**A**–**C**) The calibration curves. The calibration curves of the nomogram predicting (**A**) 3-years, (**B**) 5-years, and (**C**) 10-years OS. (**D**–**F**) Time dependent ROC curve. (**D**) ROC curves for 3-year, (**E**) 5-year, and (**F**) 10-year overall survival rates. (**G**–**I**) Decision curve analysis (DCA) plot. (**G**) DCA plot for 3-year, (**H**) 5-year, and (**I**) 10-year overall survival rates.

We evaluated the predictive ability of our nomogram by constructing time-dependent receiver operating characteristic (ROC) curves at 3, 5, and 10 years. The ROC curves demonstrated excellent discriminative capacity of our model, with areas under the curves (AUCs) of 86.9 (95% CI = 83.3–90.6), 88.4 (95% CI = 83.5–91.4), and 87.7 (95% CI = 84.1–91.3) (Fig. 6). This indicates that our model has high accuracy in predicting overall survival in parotid MEC patients.

**Figure 7:**
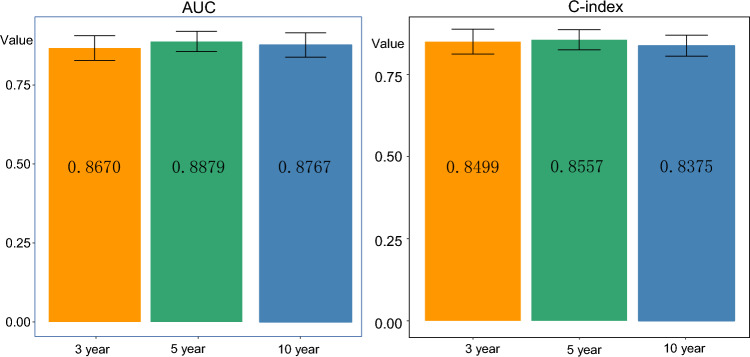
This figure presents a bootstrap analysis of a dataset, displaying the 3-year and 5-year AUC and C-index values. The analysis was performed using 1000 bootstrap replicates. The figure demonstrates the accuracy and predictive power of the model for the specified time intervals.

We also performed 1000 bootstrap resampling analyses on the dataset and generate calibration plots for the prediction model. The calibration plots showed that the curves closely aligned with the 45-degree line, indicating a well-calibrated model in practical use (Fig. 6). Furthermore, the 1000 bootstrap resamplings indicated good concordance between actual and predicted values in both the training and validation datasets, as evidenced by C-index (3-year, 0.8499, 0.775–0.914; 5-year 0.8557, 0.793–0.911; 10-year, 0.8375, 0.772–0.897) and AUC (3-year, 0.8670, 95 CI% = 0.787–0.935; 5-year, 0.8879, 95 CI% = 0.82–0.945; 10-year, 0.8767, 95 CI% = 0.792–0.947). (Fig. 7). These results further support the reliability and accuracy of our prediction model.

### Clinical utility analysis

To determine the clinical utility of our prediction model, we utilized the decision curve analysis (DCA) plot. The DCA plot illustrates the net benefit of the prediction model across a spectrum of threshold probabilities. Our model demonstrates clinical utility, as evidenced by its net benefit curve lies above both two lines across the range of threshold probabilities (Fig. 6). This suggests that our prediction model is more effective than TNM stage or grade and can aid in making clinical decisions for P-MEC patients.

In summary, our nomogram exhibited excellent predictive ability and calibration, as well as clinical utility, indicating its potential usefulness in clinical practice.

## Discussion

In clinical research, traditional linear statistical methods, such as multivariate regression and correlation analysis, often encounter challenges due to the complexity of biological variables.These variables frequently display nonlinear relationships and exhibit conditional dependencies. The fundamental assumption of these methods is that variables are independent, and there exist linear relationships among them. However, these assumptions often fall short in practice. Machine learning offers a robust solution to these problems. ML techniques can capture nonlinear relationships and conditional dependencies in data, demonstrating high accuracy and flexibility when handling clinical research data. To date, several ML-based predictive models have been developed, including models based on conditional survival forests and random survival forests for the study of advanced salivary gland cancer and primary salivary gland cancer, respectively^18,19^.The determination of prognostic factors exhibits considerable variation among patients with P-MEC, largely due to differences in pathological grading and disease stage. Furthermore, the establishment of reliable prognostic factors remains a challenging task due to limitations such as patient size and subjective grading. However, our research incorporates an expanded set of ML algorithms. Among the algorithms employed in this study, XGBoost, BSR, and LASSO screening displayed excellent overall predictive performance.

Our study identified seven independent prognostic factors and designed Kaplan–Meier plots for risk stratification, potentially providing valuable insights for clinical practice. Age, grade, T stage, N stage, radiotherapy, chemotherapy, and marital status have been determined as independent prognostic factors for the OS of patients with P-MEC.

Age, grade, T stage, and N stage are factors that have been extensively explored and validated across 18 studies published between 1968 and 2020^8^. Beyond these parameters, our study incorporates additional variables such as radiotherapy, chemotherapy, and marital status. Subgroup analyses were conducted to further understand the study population. The role of radiotherapy in treating mucoepidermoid carcinoma of the salivary glands has not been thoroughly examined in prior research. Our in-depth analysis of patients who underwent postoperative radiotherapy suggests that individuals in low-risk categories may not derive significant benefits from this treatment^20–23^. Additionally, our findings regarding LNR diverge from those of previous studies, which will be elaborated upon in subsequent sections of this paper.

A review of the National Cancer Database and a meta-analysis further confirmed the importance of age as a prognostic factor^24^. This meta-analysis of survival factors in P-MEC patients showed that in 33% of search studies, older age was associated with worse survival outcomes (HR, 1.02–6.86)^8^. A 30-year retrospective study on a predominantly MEC affected sample also demonstrated a more favorable prognosis for children compared to adults (95% ± 1.5% compared with 59% ± 0.5% (P < 0.001))^25^.

Our study found that the prognosis of P-MEC patients is grade-dependent, with poor prognoses observed for patients with high grade, consistent with prior research^8^. The inclusion of marital status in the model was supported by previous studies, suggesting that married patients may experience better prognoses due to factors such as “spousal protection”^26,27^.

Currently, the treatment of primary mucoepidermoid carcinoma of the parotid gland is based on the overall treatment strategy of parotid gland cancer, according to the National Comprehensive Cancer Network (NCCN) guidelines^27^. The role of radiotherapy as a protective prognostic factor for parotid cancer patients has been a subject of controversy^20–23^. For patients with advanced, high-grade (poorly differentiated or undifferentiated), nerve or vascular invasion, insufficient or positive margins, extraparotid invasion, or lymph node involvement, postoperative radiotherapy is recommended. Our study further analyzed the clinical value of postoperative radiotherapy at different risk levels. Compared with surgery alone, postoperative radiotherapy did not significantly improve the survival of patients in the low-risk group (P = 0.973). In contrast, there was a significant therapeutic advantage in survival performance for patients in the high-risk group (P = 0.002).This suggests that the low-risk population may not benefit from postoperative radiation therapy in cases of postoperative P-MEC, potentially preventing overtreatment. Previous studies have reported successful postoperative radiotherapy for low-grade pT2N0 P-MEC and cases where postoperative radiotherapy significantly improved survival in advanced, high-grade patients. Our results attempt to analyze the optimal population that can benefit from postoperative radiotherapy by integrating pathologic and demographic characteristics, providing limited evidence for the choice of treatment options^28–30^. Further multicenter, large sample retrospective or prospective studies are needed to validate these findings.

During surgery for parotid gland cancer, lymph node dissection and biopsy are performed. Generally, the association between LNR and OS in parotid gland cancer is worth affirming. However, due to differences in research endpoints, subjects, and statistical methods, our study suggests that LNR is not an independent prognostic factor for P-MEC patients. Q. Fang et al. reviewed more than 20 years of postoperative patients with parotid cancer and confirmed our viewpoint. Furthermore, the study believed that more than two positive parotid lymph nodes reduced the control of recurrence-free survival (RFS)^31^. A German retrospective cohort study after parotid cancer surgery also found that LNR was not the best prognostic factor^32^. Due to the failure of LNR to consider the condition of lymph nodes in the parotid gland, the study suggests that the evaluation of lymph nodes in the parotid gland should be included. Consequently, more analysis is required to determine the effect of LNR on the prognosis of P-MEC patients.

This study is subject to several limitations. Firstly, its retrospective design may lead to selection bias. Secondly, the absence of data on nerve and vascular invasion could compromise the accuracy of the findings. Additionally, the reliance on cancer data primarily from North America restricts the generalizability of the results to other regions, particularly Asia. Furthermore, both the development and validation cohorts were sourced from the same database, which might affect the robustness of the conclusions. Although the nomogram and risk stratification systems underwent comprehensive internal validation, the limited number of P-MEC patients prevented external validation, potentially limiting the external applicability of the findings. Moreover, the study did not account for all possible factors influencing patient survival, such as more detailed treatment protocols (e.g., radiation dosage, chemotherapy drugs) and more comprehensive information on individual heterogeneity (e.g., molecular markers). Despite these limitations, our clinical prediction model shows substantial potential in predicting the survival rate of P-MEC patients based on SEER database, offering reliable results for clinical decision-making.

## Conclusion

In conclusion, this study developed a machine learning-based nomogram for predicting overall survival in postoperative P-MEC patients, incorporating age, pathological grade, T stage, N stage, radiation therapy, chemotherapy, and marital status as independent prognostic factors.The feature selection capabilities of machine learning not only significantly enhance model-building process but also offer improved predictive performance and enable effective visualization of variable importance. The nomogram has the potential to aid clinicians in making personalized treatment recommendations and clinical management decisions for P-MEC patients, thereby improving patient outcomes.

### Supplementary Information

Supplementary Figures.

## Data Availability

The data that support the findings of this study are available from the corresponding author upon reasonable request.

## Abbreviations

P-MEC: Parotid mucoepidermoid carcinoma
OS: Overall survival
AIC: Akaike information criterion
AUC: Area under the curve
DCA: Decision curve analysis
WHO: World Health Organization
MEC: Mucoepidermoid carcinoma
SEER: Surveillance, Epidemiology, and End Results
XGBoost: Extreme gradient boosting
LASSO: Least absolute shrinkage and selection operator
BSR: Best subsets regression
RF: Random forest
K–M: Kaplan–Meier
IQR: Interquartile range
ROC: Receiver operating characteristic
NCCN: National Comprehensive Cancer Network
LNR: Lymph node ratio
RFS: Recurrence-free survival
