# Supplementary material for: Machine learning-based survival prediction nomogram for postoperative parotid mucoepidermoid carcinoma
Source: Sci Rep. 2024 Apr 1;14:7686. doi: 10.1038/s41598-024-58329-8 (PMC10985107; doi:10.1038/s41598-024-58329-8)
Supplement: Supplementary file 1 — Supplementary Figures. [file 41598_2024_58329_MOESM1_ESM.docx]

**Machine learning-based survival prediction nomogram for postoperative Parotid Mucoepidermoid Carcinoma**

Zongwei Huang*^1^,Zihan Chen*^1^,Ying Li^1^,Ting Lin^1^,Sunqin Cai^1^,Wenxi Wu^1^,Lishui Wu^1^,Siqi Xu^1^,Jun Lu**^1^,Sufang Qiu**^2^


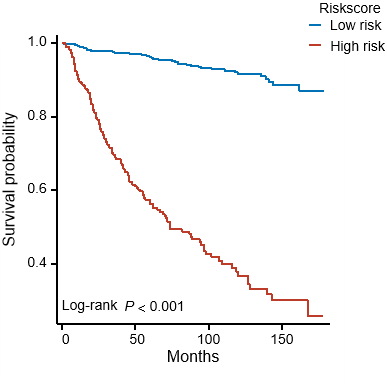


Figure S1: Kaplan-Meier Overall Survival Curves by Risk Category.


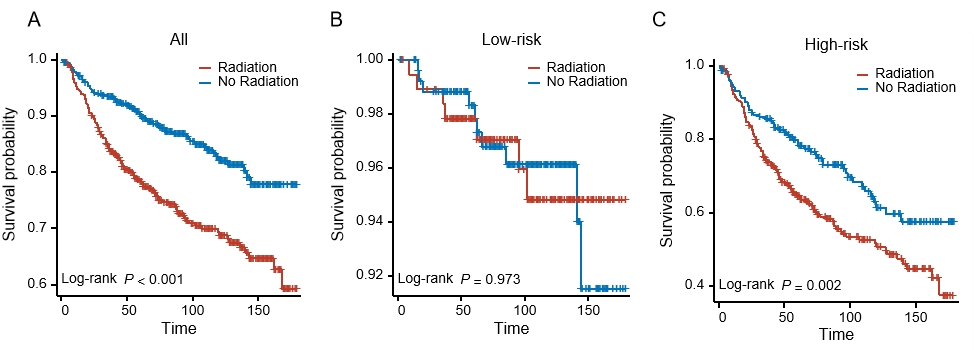


Figure S2.The Kaplan Meier curve with or without radiotherapy group.A.All: Comparison of survival probabilities with and without radiation; significant difference (p < 0.001).B. Low-risk: Radiation vs. no radiation; no significant difference (p = 0.973).C. High-risk: Radiation improves survival; significant difference (p = 0.002).


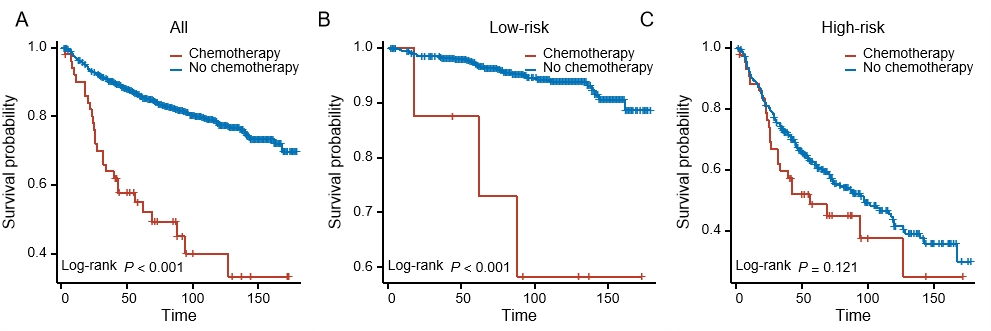


Figure S3.The Kaplan Meier curve with or without chemotherapy group.A. All patients: Survival comparison between chemotherapy and no chemotherapy; significant difference (p < 0.001). B. Low-risk: Chemotherapy vs. no chemotherapy; significant difference (p < 0.001).C. High-risk: Chemotherapy vs. no chemotherapy; no significant difference (p = 0.121).
